# Spirit of the British and American Periodicals, &c.

**Published:** 1842-04-01

**Authors:** 


### Spirit of the British and American Periodicals, &c.

### Homœopathy and Dr. Ure.

During a visit to Paris, Dr. Ure met the founder of the homœopathic system, who undertook to cure him of an obstinate constipation, by a course of infinitesimal doses. He gave him a portion of powder on the point of a pen-knife, which was to be dissolved in fifteen ounces of water, and one ounce of brandy —a teaspoonful of which was to be added to a tumbler of water. A teaspoonful of this latter was to be taken every morning, and the remainder thrown away. He did not hesitate to try the remedy, but had reason to regret following the advice which accompanied it, which was to use no remedy to remove the constipation. In the course of a few days the symptoms became so alarming, that he narrowly escaped with his life, and has never entrusted himself to a homœopathic doctor since that time.—*Pharm. Trans.* Oct. 1, 1841.

If Dr. Ure was such a goose as to trust to homœopathy to open his bowels, he was served as any reasonable person would have expected.

### First Introduction of Syphilis into Scotland.*

At the Medico-Chirurgical Society of Edinburgh, Professor Simpson read a communication relative to the history of the first introduction and diffusion of syphilis in Scotland at the end of the fifteenth century. He cited official edicts of the Aberdeen magistrates, and of the king's privy council respecting the new "infirmitez,"—issued during the currency of 1497, with various casual notices of the distemper from the contemporaneous writings of Dunbar, Lyndsay, Inglis, &c. In these early times the disease passed in Scotland under the various names of grand gore, gorre, pockis, spaynie pockis, French infirmitez, &c. Dr. S. alluded to an entry in 1502-3 in the privy purse expense book of Elizabeth, Queen of Henry VII. as containing the earliest notice of the disease in England, that he had been able to meet with. The first Scottish edicts were made with a view of arresting the progress of the disease, and ordained the application of a hot key of iron to the cheek, as the punishment of those who infringed their regulations. The Aberdeen edict was dated only four years and a month after the return of Columbus from his first voyage to Hispaniola. This edict was remarkable as being founded on the idea that the new malady disseminated itself by impure sexual intercourse—while, as is well known, it was not for several years afterwards, that this opinion of its mode of propagation, was adopted by the medical men and authors of these times. The Edinburgh regulations presupposed the disease to be communicable even by the persons that took the cure upon them, though these persons were themselves uninfected. Lastly, Dr. S. proceeded to show from the old data which he had adduced, that syphilis was new to this part of Europe, at the date of the edicts, that it was hence different on the one hand from gonorrhœa, and on the other from the leprosy of the middle ages,—both of which affections were well known in this country, before the era in question.

* London and Edin. Monthly Journ. Med. Science, Feb. 1842.

### HYPOCHONDRIASIS.*

Mr. Robarts has taken a good deal of pains with this rather ticklish subject. He tells us that the seat of the disease is in the head, the gastric symptoms being merely accidental and sympathetic: In the 1st place, Because the evidences of disease in the stomach are occasionally entirely wanting. In the 2d, Because, when present, they may be cured without producing any alleviation of the cephalic signs. In the 3d, Because they often end in those maladies which are recognised as purely cerebral, while they never degenerate, as far as his experience goes, into organic diseases of the stomach.

We think that this question is like that on the colour of the chamelion—contrary opinions are both right and wrong. One man has hypochondriasis—gradually there supervene unequivocal cerebral symptoms—and, probably, in that case the hypochondriasis had its seat in the cerebrum. Another has indigestion—hypochondriasis torments him—his indigestion is relieved, and so is his hypochondriasis. This is a common case enough, and the very same reasoning which locates the complaint in the head in one instance ought, surely, to locate it in the organs of digestion in the other. We suspect that the latter is the more ordinary case.

*Treatment.*—Find, says Mr. R., the cause, and remove it if you can. Treat symptoms, in short. Mr. Robarts is not the most stringent man in the world with regard to diet, but he denounces wine, &c. and lauds ammonia. He says —" there are certain articles that must be prohibited in all cases. These are, fermented liquors of all kinds, such as beer, wine, spirits, &c.; also spiced foods and peppers, all of which should be indulged in with great caution; they act like heat, as excitants, and thus tend further to exhaust the already exhausted powers of the brain. Here, again, we must act with circumspection. When a person has been long habituated to the free use of liquors, the sudden withdrawal of them induces a state of depression not easily overcome; yet, in every instance they should be gradually decreased, and speedily relinquished, and when necessary, medicated stimulants should be substituted. Of these, ammonia is the most generally applicable; it has a peculiar influence on the mind, which scarcely any other medicine possesses, and, I think, it may sometimes be beneficially employed. The temporary change that follows, and the suspension of the morbid train of ideas, are often succeeded by a more happy condition. It should be exhibited in small successive doses for several days. Violent stimulants suddenly administered, cause a temporary hilarity, but when their effects cease, a deeper shade of oppression ensues. But by a proper and well-timed administration of this remedy, the mind may be retained in a quiescent state for so long a period, that either the morbid ideas are lost, or only imperfectly recur."

We have found the combination of ammonia and hydrocyanic acid very useful. Sometimes it acts like a charm.

He is a *warm* advocate of the application of *cold* to the head. Cold water should be poured over it twice, or even three times a-day. Where the patient is strong, this may be done by pouring it in a stream from the spout of a kettle, or by making him hold his head under the cock of a cistern for a minute or two, while the water is running. When, however, his age or his debilitated condition will not admit of the use of so violent a remedy, a local shower-bath may be made with a moderate-sized watering-pot; and, if necessary, the water may at first be poured over the head tepid, and after a few days, reduced to a lower temperature.

---

* London and Edin. Monthly Journ. Med. Sciences, Feb. 1842.

It is essential that the quantity of sleep should be regulated. Hypochondriacs often sleep indifferently, and yet continue in bed till near the middle of the day. As a general rule, it may be said, they should not be allowed to lie in bed longer than eight hours. They should retire to rest early—say 10 or 11 o'clock—and be made to rise at 6 or 7 in the morning. In fact, they should be directed to get up as soon as they awake, even though this should be four or five o'clock.

And Mr. Robarts winds up with horse exercise.

---

### The Art of Percussion.

Dr. Bennett has contributed to our Edinburgh Monthly contemporary a long article on percussion. He is a disciple of M. Piorry, and has the lessons of his preceptor at his *finger's ends.* We shall make a note or two from his remarks.

### 1. *What hard work Percussion is.*

" In percussing, M. Piorry uses a circular ivory pleximeter, which he generally strikes with the tips of the first, second, and third fingers of the right hand, brought into a line, and pressed together by means of the thumb. The reiterated blows which it is necessary to give the pleximeter, during a careful examination of all the thoracic and abdominal organs, demand considerable physical exertion. This duty can scarcely be performed even by an expert percussor in less than half an hour; and if three or four patients are to be examined, it becomes very arduous. I have often seen M. Piorry himself much exhausted from the exertions he has undergone in this way, from visiting a single ward. But the learner undergoes still greater fatigue, from the numerous repetitions he is under the necessity of making before he is satisfied. The continual blows, also, on the tips of the fingers, (the nails being of course cut very short,) induce, at first, great pain and tenderness in them; and in my case, the skin surrounding the root of the nails became so greatly swollen and inflamed, that I was, for a time, obliged to suspend all practical examination. Indeed, those who amuse themselves occasionally by merely tapping or flipping the chest and abdomen, can form no idea of the pains and attention required to learn what may be termed the *real* art of percussion."

There is one thing which has often occurred to our own minds, as well as to those of other people, and it is this:—can such thumping and pummelling be really good for a *patient?* All our readers know that we were among the first to uphold auscultation and percussion in this country. But it is questionable whether some enthusiasts do not push it too far. The subject of it is usually one who can ill bear exposure, fatigue, or inconvenience. Yet who has not seen an out-and-out auscultator harass a poor wretch by a prolonged examination, generally useless and frequently injurious. In actual practice, and especially in private practice, a little extra nicety of diagnosis is bought, at such a price, too dear, and we believe that those who attempt it will not uncommonly find it so. It may be very gratifying to a physician to be able to draw a map of his patient's bowels, and we cannot but think that, in some instances at all events, the gratification will be heightened by the very procedure tending to afford an opportunity of verifying it.

*The Hammer.*—Dr. Bennett uses the plexor of Dr. Winterich, of Wurzburg. Considering that percussion is such very hard work, it must be owned that he is right. He employs an oval pleximeter. Its length is two inches, and breadth one inch. This pleximeter may be applied with great precision to every part of the chest, even in emaciated subjects. It may be made of ivory, wood, or metal. An inch-and-a-half scale drawn upon the surface, will be found useful in measuring the exact extent of the dulness, and determining the differences which may occur in it from day to day.

*The Gamut.*—" M. Piorry considers that nine elementary sounds are thus formed, which he has designated, from the organ or part which originates them, '*femoral, jecoral, cardial, pulmonal, intestinal, stomacal, osteal, humorique,* and *hydatique.*' I consider that all these sounds may be reduced to three elementary ones: that, in point of fact, there are only three tones occasioned by percussion, and that all the others are intermediary. These three tones are respectively dependent, 1*st*, On the organ containing air; 2*nd*, On its containing fluid; and, 3*rd*, On its being formed of a dense uniform parenchymatous tissue throughout. These tones, therefore, may be termed the *tympanitic,* the *humoral,* and the *parenchymatous.* Percussion over the stomach gives the best example of the first kind of sound; over the distended bladder, of the second; and over the liver, of the third. The terms jecoral, cardial, pulmonal, intestinal, and stomacal, however, may be used to express those modifications of sound produced in percussing respectively the liver, heart, lungs, intestines, and stomach."

*The Sense of Resistance.*—A percussor has much to learn on this point. For, by the sense of resistance, is understood the peculiar sensation resulting from those impressions which are communicated to the fingers on striking hard, soft, or elastic bodies. It is of the greatest service in determining the physical condition of the organ percussed. The sense of resistance bears relation to the density of the object struck,—hence firm and solid textures offer more resistance than those which are soft or elastic. The sense of resistance should be as much educated by the physician as the sense of hearing, and it would be difficult for an individual, practised in the art of percussion, to say which of these two points is most valuable to him.

*How to map out an Organ.*—In percussing any particular organ, the pleximeter should be first applied over its centre, where the sound and sense of resistance it may furnish, are most characteristic. Two blows with the hammer are generally sufficient to determine this. From the centre, the pleximeter should be moved gradually towards the periphery, or margin of the organ, and struck as it proceeds with the hammer, now forcibly, now lightly, until the characteristic sound of the next organ be elicited. The pleximeter is then gradually to be returned towards the organ under examination, until the difference of tone and sense of resistance become manifest. In this manner having first heard the two distinct sounds well characterised, we shall be better enabled to determine with accuracy, the limit between the one and the other. This may be done exactly after having determined, whereabouts the line of separation is, by placing the long diameter of the pleximeter transversely across it, and striking, first one end of the instrument, and then the other, till the precise spot is determined. This spot should now be marked, by placing with a pen a dot of ink on the skin. The opposite and then other portions of the margin of the organ should be limited in the same manner, and these in turn should be marked with dots of ink, until the whole organ be completely examined. Then by uniting all these dots with a line of ink, we have the exact form of the organ drawn upon the skin. When it is thought necessary to render the first mark permanent, in order to see if any subsequent change take place in the size of the organ, or extent of the dulness, it may be rendered so, by carrying lightly a stick of argent. nit. over the ink line, while it is still moist.

We should conceive that did he go to work in this way, a physician in good practice would find twenty-four hours short allowance, for the day. Surely such recommendations as these are the transcendentalism of practical medicine.

Far be it from us to disparage any attempt at nicety of diagnosis. But we repeat that it may be bought too dear. Patients, even hospital patients, in this country, cannot be treated as machines or dead bodies. It is otherwise in France. There, any favourite mode of exploration, however fatiguing, however disgusting, creates no check from the feelings of either patients or bye-standers, and the unhappy creature, who has been pummelled by a Piorry in the morning, may be exposed by a Lisfranc in the afternoon. More moderation is requisite in England.

---

### Midwifery in China.

Dr. Churchill has published, in the last number but one of our Dublin contemporary,* an interesting translation of a Chinese Treatise on Midwifery by Dr. Lockhart, who, it appears, was sent into China a few years ago by the London Missionary Society, in hopes that medical science might prove a means of establishing a freer intercourse with the people of that country. We cannot say that this work inspires us with any very high idea of the state of obstetric science among our celestial *confrères*. We shall extract a few passages from it.

After some prefatory remarks, in which the author insists that this Treatise should be in the hands of all classes, but more especially of the rich, who, he says, "are proud and will not listen to advice," we come at once to the subject of—

*Parturition.*

"Six characters comprise the whole business: sleep, bear the pains, slowly come to the tub."†

"It is of no consequence whether it be late or early (since the commencement of labour); but she must not go too soon to the tub or use efforts, and she must not listen to what some midwives say, that the head of the child presents externally, causing her to go at once to the tub, for this would mar the whole of a business, which is ever completed by nature alone. If at the proper time the fœtus be able to work itself out, what need is there for being hasty? because it is to be feared, that as the strength of the fœtus is but small, if, when it turns its body, this strength be exhausted, it is brought to the gate of life, but cannot work itself out. Still there is a time when it is proper to make a slight effort, and then one exertion will be sufficient to cause the fœtus to lose its place and descend. Thus at the set time the melon ripens and the tendril breaks. At this period the blood and breath are dissipated (partial recovery), the bones and joints of the body are loosened for a moment, a gush of water takes place, and without effort the child is born, and the mother herself does not know how it came about. Some say, that as we make an effort in passing an evacuation from the bowels, why not do the same in labour? But they do not consider that the fæces are mere dead matter, and therefore an effort is required, but the fœtus is able to turn itself, and the time that it does so must be waited for; not only is the use of effort needless, but it is absolutely injurious; for the fœtus sits upright in the womb till the time of birth, when it bends down its head and turns its body towards the contracted opening of the womb patiently, till it turns its body to the gate of life, the head being then below and the feet above, as though it were suspended upside down, it comes out at once. If the fœtus has not yet turned its body, and efforts be made to expel it, then the feet will come out first;

---

* Dublin Journal, Jan. 1842.

† In China, when the strong bearing-down pains come on, the woman is supported in a half-reclining posture, and a tub or deep wooden platter placed so as to receive the child on its expulsion.

this is a strange occurrence, and an elegant name is given to it; viz. 'the foot-treading or water-lily birth.' If the turning of the body be not quite complete, and an effort of expulsion be made while the fœtus lies transversely in the womb, then a hand will come out first, and this is called the 'begging-salt birth.' Again, if the body be turning, and yet not quite straight, and efforts be made too early, the axis of the body being directed to the right or left, the hip or breech will present, but cannot come out; and all this arises from exertion being used before the proper time. All persons should be earnestly exhorted on no account to use improper efforts; at the same time efforts are not altogether to be discarded; but when used, it is only to be for the period occupied in drinking a cup of tea: above this all irregular exertion is to be avoided, for, as in the evacuations, if the fæces be not prepared, whatever efforts be made they will not come out, how much less in bringing forth a child. Some one may ask how is this swallow-of-tea time to be known, that efforts may be made? The time is certainly not always the same, if the fœtus be pressing downwards to the gate of life, then the bones and joints of the woman's body are relaxed, the chest bears down, the abdomen becomes distended or tense in a remarkable degree, the evacuations pass involuntarily, scintillations, as of golden flowers, are seen darting before the eyes, this is the precise moment, and if she be now brought to the tub, some efforts made, the mother and child will be separated; where lies the difficulty in this?"

The Chinese doctor appears to entertain a most holy horror of midwives. Generally speaking, he says, these people are stupid and ignorant, and not acquainted with the principles of things; immediately they enter the door, instead of inquiring, whether the stage be early or late, or whether the pregnancy be complete or not, they order the woman to sit on the grass and to make efforts, insisting that the child's head already presents, and they tell her to twist her loins; they rub her abdomen, or they introduce the hand into the vagina and endeavour to feel the child, this is highly injurious and hurtful, and arises altogether from a desire to appear to be doing something, unwilling to allow things to go on quietly. There is also another class of perverse women, who take advantage of this time to quarter themselves upon persons for the mere sake of gain. The evil of this is by no means slight. It is said, that between the Woo and Yue countries, these women are called 'secure females', between Keary and Hevery they are called 'receiving-to-life' females; between We and Ning they are called 'welcome life women.' Regarding the meaning of these two words, *welcome* and *receiving*, they are used because these old and experienced women are directed to receive the child as it comes down, and to take it up and to put it on the bed, but they are on no account to pull it by the arms or legs.

*Illustrative Cases.*

The celestial author gives several cases in illustration of his style of treatment, which consists in trusting to nature. One of these is the following.

"Some time ago, Pofa, the secondary wife of Mr. Chang, who lived at Hosham, a woman of tender years, though fully formed, when she became pregnant, labour came on at the eighth month; she had severe pains for several days, and the child was born, but shortly died; she again became pregnant, was prematurely delivered, and the child died as before. I then said, that they must inform me when she was again in labour. The next year, at the eighth month, she was again in labour, which continued for three days, without success; when, suddenly recollecting my words, they sent off post-haste to call me: on the road I met the coachman, who said he was going to call her parents to take a final leave of her; when I arrived it was already night; on feeling her pulse I found it was not yet at the lowest ebb, and that she was still healthy. The midwife, on being questioned about the affair, said that the child's head presented but could not come out. I ordered her to be put quickly to bed, and not to be disturbed, giving her at the same time a little medicine to soothe the womb. The next morning her husband came to me laughing, but did not speak. I asked the state of matters, and he said all is well. Yesterday, said I, the child's head was at the gate of life, how is it now? He answered, it has disappeared, and, with a loud laugh, went off. 120 days after this, that is *at the term of twelve months after conception,* she was delivered, and they said I was the child's father (ascribing the merit of saving its life to me), and it is now eight years old. And now I come to know, that on former occasions, they had pulled out the child by force, and it was only because the woman was young and strong that she survived."

*After-Treatment of Parturient Women.*

"After labour let the woman be put to bed, let her have a high pillow to lean against, so that she may not lie flat down; the knees are to be raised and not to be stretched out; and let her immediately drink a cup of boy's urine warmed; she ought to shut her eyes, and be kept quiet; but she must not sleep soundly, for should she be very weary and sleep soundly the blood and heart will flow upwards and occasion dizziness of the head; she must not be spoken to in a loud tone or in a hurried manner, lest she be alarmed; she must be protected on all sides from the wind; she must not be asked whether she has pain or not; she must constantly use boy's urine mixed with warm wine in equal quantities, taking a cup at each time, so that in one day three or five cups are taken."

"Whether a boy or girl be born, is according to the husband's fate, for the sacrifices of a hundred generations depend on the husband's family; how can it result from the family of the wife? if daughters be successively born, this is a common occurrence, why should it cause distress and grief, and produce painful feelings? when we see a stupid husband taking offence against his wife and making himself ill, shortening his days, how very ridiculous and absurd this is; it is far better on all such occasions to be placid and liberal minded: there are even some who disown their daughters; those whose cruel hearts thus act contrary to the principles of reason must ever be haunted by future calamities. The after-treatment varies according to the custom of different places. In some parts they use red sugar, in others shon chachoo yu, an acrid plant, a drink made of pepper boiled in water; but there is nothing better than the hot wine and boy's urine; but should there be severe pain in the bowels, the use of sang hiva soup will be found very beneficial."

Boy's urine seems to be a very favourite remedy in China, the Doctor repeatedly insists upon its use. This, however, is not so bad as some of the prescriptions we meet with a little farther on; we should advise the adoption into the next edition of our Pharmacopœia of the following—

*Remedy for Prolapsus Ani in Children in consequence of Dysentery.*

"Take a little dry fæces, *you must not use such as are found in the roads, but they should be from unhealthy persons,* pulverize it carefully, and mix it with rice and water; two doses will effect a perfect cure."

The most extraordinary cure, however, is to come.

"In the eighteenth year of Kea King (1814), Chow Ping Han, the Han Hoe magistrate of Canton, a literary man, had an infant son, who, when he was a month old, became very much diseased in all parts of his body; the father seeing this, vowed to Heaven that he would print and publish forty copies of this Manual of Midwifery, and, *wonderful to relate,* the boy instantly became perfectly well, and enjoyed good health!!!"

Wonderful indeed!

### Observations on the Incipient Stage of Cancerous Affections of the Womb. By Dr. Montgomery, Dublin.

[Dublin Journal.]

All observations on obstetric subjects from Dr. Montgomery are sure to command the attention of the profession, characterized as they always are by practical information and clear judgment. The disease in question is one of the most destructive, painful, and loathsome to which the female sex is liable. When confirmed, it defies all remedies, including the knife; but when early detected, our author thinks it may be often arrested in its course, and its victim rescued from a torturing death.

"I am satisfied, that there is a stage of cancer uteri which precedes the two usually described by authors; a stage, in which, the nature of the disease may be detected, its further progress arrested, and its germs destroyed, and the reason why this stage is not more generally recognized is, that the accompanying symptoms are frequently so slight as to attract very little the attention of the patient, and thus, are suffered to remain without treatment, until a profuse hæmorrhage, or some violent fit of pain sounds the alarm, and then, on examination, the disease is found to have passed into its second stage; the surrounding tissues are indurated and consolidated with the organ concerned, and no human means hitherto discovered can do more than blunt the thorns thickly strewn along the path, which the sufferer must tread, to 'the house appointed for all living.'"

*Symptomatology.*—Sharp, but fugitive and lancinating pains in back and loins—across the supra-pubic region or along the front of the thigh—sometimes in the direction of the sciatic nerve, producing numbness, or debility of the limb. In a large proportion of cases, there will be found a decided fulness, or even a distinct tumour in one or other iliac fossa, with fixed pain, tenderness on pressure about the inguinal ring—irritation of bladder—dysuria—sensation of piles about the rectum, &c. The menstruation is sometimes disturbed, often quite regular—occasional bursts of hæmorrhage—little or no leucorrhœa—unimpaired appetite (till the complaint has lasted some time)—disturbed sleep—flabbiness of flesh—pallor of countenance.

*Examination.*—Margin of os uteri hard, and often slightly fissured, projecting more than natural into vagina, irregular in form. "In the situation of the muciparous glands, there are felt several small, hard, and distinctly defined projections, almost like grains of shot, or gravel, under the mucous membrane. Pressure on these, with the point of the finger, gives pain, and the patient often complains that it makes her stomach feel sick.

The cervix is, in most instances, slightly enlarged and harder than it ought to be. The circumference of the os uteri, especially between the projecting glandulæ, feels turgid, and to the eye, presents a deep crimson colour, while the projecting points have sometimes a blueish hue."

This stage is very slow—being sometimes spread over years, before the second stage sets in.

*Pathology.*—Dr. M. is convinced that, in a great majority of cases, the first morbid change takes place in and around the muciparous glands which exist in such numbers in the cervix and margin of the os uteri, which become indurated by the deposition of scirrhous matter around them. They feel at first like shot or gravel, and afterwards become unequal, bumpy, or knobbed, like the ends of one's fingers. This is the *second* stage of the disease, though usually described by writers as the *first*. It is irremediable.

*Diagnosis.*—The "IRRITABLE UTERUS" is the only complaint which can be confounded with the one under consideration. The *former* never produces organic changes—the *latter* does. The *former* is therefore unaccompanied by any enlargement of volume in the parts, which always accompanies or soon follows the disease now treated of.

*Treatment.*—Local leeching or cupping, with anodyne fomentations, form the first step in the treatment. Mercury, in some form or other, is almost indispensible, so as to bring the system very gently, but decidedly under its influence. It may be combined with very small doses of iodine, in conjunction with camphor, opium, hyoscyamus, or cicuta. "Afterwards, iodine or hydriodate of potash may be used both internally and externally; and iron will be found a most beneficial and powerful agent, especially in the form of the saccharine carbonate, or the carbonate given in the nascent state.

The iodide of iron, which combines, to a certain degree, the powers of both remedies, may also be used with advantage in most cases, and will be best administered in the form of Dupasquier's syrup, which is now prepared, of different strengths, by our chemists and apothecaries."

Our author has observed marked benefit result from the exhibition of arsenic in this dangerous complaint. Counter-irritation, the half-bath, injections of warm water into the vagina—and, when congestion is removed, and morbid sensibility only remains, anodynes added to the injections will be serviceable.

Every kind of irritation and excitation should be sedulously avoided, and the most mild and abstemious diet enjoined.

Although this is the stage in which amputation might be successful, yet our author does not recommend it, "because the operation is a very formidable one, and he knows the affection to be curable without it." This is a very substantial reason for eschewing the operation certainly.

We have thus condensed the valuable matter of this communication of Dr. M.'s, and have no doubt that it will prove acceptable to our readers. Some cases in illustration are added; but we are unable to comprehend them in this brief article. They are highly deserving of perusal by the medical practitioner.

---

### CASE OF ACCIDENTAL POISONING BY ARSENIURETTED HYDROGEN. By Dr. O'REILLY, of the King's and Queen's College of Physicians, &c. [Dublin Journal.]

It is but very rarely that death is produced by this mode of taking arsenic. though the material itself is one of the most deadly forms of the mineral. There are but few records of poisoning by the inhalation of arseniuretted hydrogen—one was in the person of Gehlen the chemist, and the other that of Mr. Beard, a young British lecturer on Chemistry.

On the 23d October, 1841, Mr. Brittain, a druggist and chemist, aged 31, inhaled, at two different periods, about 150 cubic inches of impure hydrogen gas, believing that, if pure, it would not be injurious. But, immediately after the second inhalation, he was seized with giddiness and faintness, followed by shivering and an evacuation from the bowels, and two ounces of blood from the urethra, without pain. These were followed by pain in the lower extremities, and numbness of the superior, with tingling sensation. These phenomena continued for two hours, when they subsided, and Mr. B. was seized with pain in the loins, vomiting, &c. which continued some hours. When Dr. O'Reilly arrived, there was nothing remarkable in the patient's appearance. He complained of great weakness—bitter taste in the month—pulse 90, and feeble—surface cool—voice very low—dull pain in the epigastrium. A draught of ammonia and laudanum was prescribed, to be repeated every three hours, with pediluvium and emollient injections. At ten o'clock the same night, eight leeches to the epigastrium. Four drops of the black drop, with three grains of hyd. cum creta, every two hours.

24th. Vomiting continued through the night—bowels cleared by the enema—no urine—face of a copper colour—body of a greenish hue—pulse 80 and strong—still some tenderness at epigastrium—troublesome hiccup. The same remedies, with diluent drinks.

On the 26th we find the jaundice disappearing—the bowels freely evacuated—discharges loaded with bile—no urine—still some vomiting. Salines with carbonic acid—chicken broth—wine and water. 27th. Still no urine. The jaundice has disappeared—no fever—no pain—bowels open—face rather œdematous—ammoniacal odour on the breath—somnolency. The hip-bath prescribed—diluents—nitre and nitrous ether, &c. 28th. Deep irregular ulcer on the tongue—breath ammoniacal—no urine in bladder when catheter was introduced. Symptoms got rather worse, and he died on the evening of the 29th.

*Dissection, 36 hours after death.*—"Universal anasarca; integuments of the abdomen of a slight greenish colour, particularly at the sides; abdomen greatly distended with gas; on opening the chest, we found the lungs completely collapsed, natural in structure, but containing little air; a fluid, to the amount of two pints, of a reddish-brown colour, without odour, was effused into the chest; heart, pale and flabby, not containing blood, nor changed in structure; a little fluid in the pericardium.

*Abdomen.*—Liver of a deep indigo colour, not increased in size; the gall-bladder distended with bile; the kidneys of a deep indigo colour all through, the left particularly large, the internal structure resembling much the spleen in appearance; the right smaller and firmer; the stomach empty; two distinct patches of inflammation were observable in the greater curvature; the mucous membrane easily separated; the bladder empty and natural.

*Head.*—The dura mater healthy; arachnoid somewhat vascular, containing air bubbles underneath; the substance of the brain bloodless; no fluid in the ventricles."

We cannot follow Dr. O'Reilly through the course of his manipulations: it is sufficient to say that arsenic was unequivocally detected. But which of the ingredients?—the zinc or the sulphuric acid? Six specimens of the latter, obtained from different sources, were subjected to trials, and "all were found to contain large quantities of arsenic."

For various observations and reflections, curious, scientific, and useful, we must refer to the original paper in our respected contemporary.

---

### CASES ILLUSTRATIVE OF DISEASE SEATED IN THE CEREBELLUM. By W. JACKSON, Esq.

[Provincial Journal, Dec. 1841.]

*Case* 1.—Mr. R. æt. 25, married. Came under the care of Mr. Jackson, 20th August, 1830. About twelve months before this he became subject to paroxysms of severe pain in the head, which was at first confined to the upper and anterior part, but for the last four months had become fixed (during the fits) in the upper and back part of the head and neck, shooting forwards to the forehead, the face was flushed, and the head steadily inclined forwards. The attack was often occasioned by rising from bed in the morning, and usually lasted for two or three hours. The pain was of the most agonizing kind, and was described as if something were darting downwards along the spine. When lying in bed, and frequently when in a sitting posture, Mr. R. would fancy himself falling down, not only during the paroxysm, but also during its absence. The pulse was generally unaffected during the intervals, but varied considerably at other times, being sometimes rapid and almost imperceptible, and again slow, being once reduced as low as 40. The exciting cause of a fit appeared, on many occasions, to be muscular action succeeding to a state of rest; the proximate, the consequent determination of blood to the seat of the disease; but any violent mental emotion would occasion an attack. At one period the attacks were accompanied by a copious discharge of flatus from the stomach, with decided relief, and when the attack was more severe than usual, vomiting often supervened. The paroxysms, which usually ceased suddenly, were generally followed by perfect ease. Several of these attacks would frequently occur during the night, and in the intervals more or less sound sleep be obtained. As the disease advanced, vision became considerably impaired; the other senses, however, remained perfect to the last. His general health was unaffected, excepting that the stomach became sometimes deranged in its action.

From the general character of the attacks, Mr. Jackson, as also Dr. Knight, who attended with him, were inclined to regard the case as of a mixed nature, presenting many of the features of a nervous or hysterical affection, but also leading to the suspicion that serious disease existed within the cranium.

The treatment adopted consisted, at first, under the supposition of its being more or less inflammatory in its nature, of local bleedings, counter-irritation, and rest. Afterwards, perceiving that as debility increased the symptoms became aggravated, tonics, improved diet, wine, change of air, and the shower-bath, were ordered with much benefit. Under this treatment his strength improved, and the attacks became much less frequent and less severe: on the 13th of October, however, after a journey to Leeds, a severe and unexpected attack came on, gradually increasing till a state of partial insensibility supervened, and, seemingly exhausted, he sank in two hours, without suffering any spasmodic movement.

*Post-mortem Appearances.*—The veins of the pia mater were turgid with blood. The substance of the brain likewise was more vascular than usual. The ventricles were distended with a clear fluid. Several parts in the interior and at the base of the brain were softened, especially the fornix, tractus opticus, hyppocampi, and tubercula quadrigemina. The right pes hyppocampi was enlarged, and in its interior was found a tumor of the size of a large pea, of a whitish grey colour externally, and presenting a similar appearance internally. The cerebellum was softened, and in the centre of its left lobe was found a globular tumor, of firm consistence, one inch and a half in diameter. The general aspect of this tumor resembled those found in scrofulous subjects. The effusion into the ventricles had occurred, no doubt, during the last attack.

*Case* 2.—George Beighton, æt. 44, married, applied to Mr. Jackson, May 17th, 1841, having been ill then for one month. He complained of constant vertigo, which continued throughout his disease; he was unable to walk except in a very unsteady manner; and suffered from severe pain referred principally to the back part of the head; pulse 90, full; tongue white. He was ordered to be bled and purged, and a blister was applied to the back of the neck, without relief. In a few days severe vomiting came on, which continued throughout his illness; the pain in the head became more urgent, returning in distinct paroxysms, which would be occasioned by the slightest movement of the body, to prevent which, he constantly kept his hands fixed firmly upon his head, which was brought forward upon the chest. He carefully kept his eyes closed. The intellectual powers were not at all impaired; nor was the strength materially affected. The patient frequently expressed a feeling as if he were on the point of falling from a considerable height. These paroxysms of pain became more and more severe, in spite of all the remedies employed, till at last the patient, apparently worn out by the severity of his sufferings, and conscious to the last, sunk on the 5th of June.

*Post-mortem Appearances.*—At the point where the occipital bone joined the parietal, the dura mater adhered firmly to the arachnoid. The ventricles were filled with fluid. The cerebrum generally was natural, and of its usual firmness.

The cerebellum externally presented increased vascularity; its right lobe was healthy throughout. The left lobe however was softened, and appeared in a disorganized state; on cutting into it, it was found that nearly the whole of this lobe was the seat of a cavity filled with a sero-gelatinous fluid. There was no lining membrane, but the sides of the cavity, consisting of the substance of the cerebellum, were quite softened.

This case presented the appearance of an acute disease, while the former exhibited an example of the more chronic form.

*Case* 3.—J. G., æt. 68, was suddenly seized in the Spring of 1838, with extreme vertigo, temporary insensibility, and extreme coldness of the whole body; pulse slow and labouring, afterwards feeble. He continued in an apparently exhausted state for two or three hours, vomiting occasionally. The symptoms which remained two days after the attack, were extreme vertigo, pain in the occipital region, and considerable unsteadiness in his muscular movements, so as to resemble, in walking, a person who is slightly intoxicated. He retained perfect possession of his intellectual powers, and, beyond this unsteadiness, there was no paralysis. He was cupped and repeatedly blistered; kept on low diet; and advised to remain at rest for some time. His restoration was partial, and very gradual from the first seizure to the period of the second attack.

Between two and three months after this he was seized, shortly after a hearty dinner, with sickness and vertigo, and shortly afterwards became nearly insensible, his extremities being extremely cold; warmth having been restored, he was bled to eighteen ounces, and a large dose of calomel administered. On this occasion, the most remarkable symptom present was, the perfectly unsteady and unmanageable state of the muscular powers. In six hours' time, he became insensible, and expired soon afterwards.

*Post-mortem Appearances.*—The cerebrum was generally in a healthy state, excepting that the ventricles were somewhat distended with serum. The branches of the basillary artery were thickened, opaque, or whitish-brown; their coats were easily broken down, and felt as if they contained a quantity of coarsely powdered chalk. The interior of each lobe of the cerebellum contained several dark coagula of blood—one, especially, surrounded by a cyst, as if of older date. Around those coagula the structure of the cerebellum was in a completely softened state, as, indeed, was more or less the whole substance of that organ.

*Remarks.*—The functions of the cerebellum are as yet but very imperfectly understood; these cases, however, as far as they go, are certainly much in favour of the views of M. Fleurens—that the cerebellum gives to the muscular system a general harmony of action, and a precision of purpose; and that an impairment of its function is attended by agitation, unsteadiness, and irregularity of muscular action.

Is the cerebellum in any way connected with sensibility? Mr. Jackson is inclined, from these cases, (especially the second,) to answer in the affirmative. The difficulty consists in distinguishing the effects produced by the disease in the cerebellum, from those arising from the affection of the contiguous structures, more especially the membranes.

It is highly probable that pathology will, in time, do more to explain the real nature of the functions of the cerebellum than experimental physiology, which is liable to two great objections, viz. the great shock produced by the operation, and the necessity of involving other structures.

### Case of Closure of the Os Uteri, which required an Operation. By T. R. Pugh, Esq. Salem.*

Mrs. ——— came under the care of Mr. Pugh on the 10th of June, 1840, apparently suffering from an attack of dysmenorrhœa. It appeared however that she had not menstruated for two years, and that there had been no discharge of any kind from the uterus since she had her last child, which was about two years before. The usual remedies for dysmenorrhœa were tried without effect; and as there seemed to be much enlargement about the abdomen, and her symptoms became alarming, Mr. Pugh determined on puncturing the uterus, which was performed on the 22d of June, in the following manner: a middle-sized scalpel, wrapped with fine calico, was introduced upon the index finger of the right hand to the indentation left by the closure of the os tincæ, and the point was then gently forced through the coats of the organ, about two pints of fluid immediately escaped, and this was attended with considerable relief; in the course of the next twelve hours, two pints more flowed away. A gum catheter was then introduced, which remained in until the wound was healed and the orifice formed. She recovered her health rapidly, and has continued well except a mild attack of leucorrhœa. Menstruation has been quite regular ever since.

It seems that immediately after the delivery of her last child, she was attacked with inflammation of the neck of the womb; the lochia never appeared. During the two years that she remained ill she always suffered from severe pain at the time of her monthly period, and occasionally had vicarious discharges from the anus or lungs.

---

### Case of Aneurism of the Arch of the Aorta, pressing upon the left Bronchus, &c. By Robert Spittal, M.D.†

The subject of the following observations, a gentleman of the legal profession, æt. 47, unmarried, and somewhat irregular in his habits, had for several winters suffered from bronchitis. On the 18th of October, 1840, he again came under the care of Dr. Spittal. On examining the thorax, a rounded tumor, measuring between two and three inches in diameter, and of an elevation of about half an inch, was observed a little above the centre of the sternum, in the mesial line. The sternum was distinctly perceptible over the tumor, and continued to be so till the death of the patient; the integuments, at first of a normal appearance, subsequently became reddened, presenting very much the appearance of the skin over an abscess.

There was a considerable heaving impulse in and around the tumor, the sound on percussion over it was dull, as was also the case to the left, over nearly the internal two-thirds of the clavicle; the dulness extended down below the second rib of this side. The respiratory murmur was absent, where the percussion was dull: but distinctly audible over the rest of the chest. At this time there was frequent cough, with yellowish tenacious expectoration, especially troublesome at bed-time, and accompanied with slight acceleration of the breathing, which occasioned much annoyance to the patient.

These symptoms continued much the same, with the exception of the catarrh becoming more severe, until the latter end of November, when, after exposure to cold in the open air, the right lung also became affected with bronchitis. On the 26th of this month, he was found by Dr. Spittal labouring under very

---

* The Maryland Medical and Surgical Journal.

† London and Edinburgh Monthly Journal of Medical Science. Jan. 1842.

considerable aggravation of all his symptoms. The pulse was 120, of increased strength—skin hot and dry—respirations 40 in the minute—catarrhal râles, chiefly mucous, increased in the right lung, while scarcely any respiratory murmur was perceptible over the left, and then only on coughing or on forcible inspiration. Œdema of the feet and ancles, with lividity of the face, and slight apparent protrusion of the eyes, now appeared, and the patient became unable to maintain the horizontal position, except for a short period.

After the exhibition of antimonials, and the free application of blisters, slight relief was obtained. On the 30th, however, the dyspnœa increased—pulse became more rapid and feeble, in spite of stimulants—face and upper part of the trunk slightly livid—increase of œdema of lower extremities, and he gradually sank and died early on the morning of December 1st, 1840.

The sounds perceived in the region of the tumor were similar to those in the præcordial region—which were normal—except on one or two occasions during the earlier part of the attack, when a short and sharp murmur was heard, synchronous with the first sound of the heart. The patient thought he observed the commencement of the tumor about half a year before Dr. Spittal's attention was directed to it. He likewise stated that, about two years before this, he had an acute "cutting" pain under the sternum, but had had little or none since.

The treatment, apart from that applicable to the acute pulmonary attacks, was directed to alleviate the symptoms arising from the aneurism, and to hinder as much as possible its increase. With this view the ordinary means of rest, both mental and physical, were enjoined; with at the same time absence from all unusual stimuli: and a very moderate diet was ordered.

*Sectio Cadaveris,* 36 *hours after death.*—On opening the thorax, a large sacculated aneurism was found to arise from the anterior and a little to the left side of the aorta, about an inch above the semilunar valves, involving likewise the whole of the lower and anterior part of the arch, which was very much dilated. The aneurism extended from the upper part of the sternum to about the level of the cartilage of the third rib; the sac was firmly adherent to the edge of the sternum throughout the whole length and breadth of the part described, the latter having served as part of the anterior parietes of the tumor. At several places the bone was quite exposed internally, at some hollowed out and quite diaphonous, especially at the upper and left side, near the sterno-clavicular articulation.

The interior of the tumor was quite irregular from rounded elevations, the normal structure of the artery becoming obscured or lost immediately at the commencement of the dilatation. The great vessels arising from the dilated arch were all pervious, but at their origin were somewhat thickened and of a reddish colour. About one-third of the sac was occupied by numerous layers of fibrine of a pale ash-colour.

The right lung had its air-tubes loaded with thin and frothy mucus, and was slightly emphysematous. The left lung presented the various stages of pneumonia. Immediately below the bifurcation of the trachea, five of the cartilaginous segments of the left bronchus were more or less completely exposed, and were somewhat flattened.

*Remarks.*—From the first there could be no doubt as to the nature of the disease, which probably commenced at the period when the "cutting" pain was first felt, although no tumor showed itself externally till about six weeks before his death; its progress internally, where alone it extended, was clearly manifested in the occurrence of pressure on the neighbouring parts, and it seems evident that, had the patient lived, the aneurism would have burst into the left bronchus. As it was, the patient sank under the combined effects of obstruction to the respiration and circulation, as the aneurism must, from its position during life, have pressed upon the vena innominata and left jugular, on the descending vena cava for some extent, and on the left pulmonary veins, together with the effects consequent on the morbid state of the lungs described.

Perhaps the most interesting point is the impediment which the mechanical obstruction of all respiration in the left lung placed in the way of the ordinary means of diagnosis, denying us altogether the aid of any indications of a respiratory or vocal character, and limiting us to those afforded by percussion and the general indications. The total absence also of the characteristic sputa, formed an additional bar to the diagnosis of the inflammatory condition of the left lung.

---

### Paraplegia.—Abstract of a Paper read before the St. George's Hospital Medical and Surgical Society. By Athol Johnson.

The subject of the present paper is paraplegia, or palsy of both sides of the body, generally affecting only the lower extremities, in contra-distinction to hemiplegia or palsy of only one side; and I have selected it as being a disease still but imperfectly understood, and therefore the contribution of any new facts may perhaps be attended with some advantage. Dr. Baillie, in a paper on the subject, published many years ago, observed, that he thought the disease had increased in frequency within the few previous years. I know not whether this be true or not, but certainly, within my very limited experience, I have had an opportunity of seeing at least fifteen or twenty cases, and have assisted or been present at the post-mortem examination of seven or eight.

Paraplegia is, of course, not a disease in itself, but merely a symptom of some morbid affection occurring in some other part of the body, and which is usually chronic in its nature.

These morbid affections or causes may be classed under the following heads:—

1st. It may arise from disease of the vertebræ, especially caries.

2nd. From injury of the vertebræ or spinal cord.

3rd. From other causes.

Of the first two I shall not speak at all here, as they are well understood, and of frequent occurrence.

Of the "other causes," two great divisions may be made, namely, those which are *functional*, and those which are *organic*.

That paraplegia may be functional, is proved by the action of certain poisons upon the body, such as the Wourara poison: when this is administered to a dog, the animal first loses sensibility, then the hind legs become powerless, afterwards the fore-legs, and finally the ears. As the effects of the poison begin to wear off, the dog first regains power over the ears, then over the fore-legs, but the hind-legs remain paralyzed for some time after;—in fact, the animal is paraplegic. At last this also disappears, and the dog is perfectly restored.

The *functional causes* may be farther subdivided as follows:—

1. Over-excitement of the nervous system, as in the case of professional persons, or those obliged to use great mental exertion.
2. Inflammation of the membranes of the spinal cord.
3. A peculiar state of the general system, existing usually in young females of a chlorotic habit, or what has been termed "hysterical paraplegia."
4. Great demands upon the nervous system; as excessive venery, indulgence to a great extent in ardent spirits, &c.

The first of these, though not met with in hospital practice, is, I understand, not very uncommon among literary persons, especially lawyers: its inorganic nature is proved by the speedy cure effected by rest, abstinence from mental exertion, and change of air.

A case of paraplegia produced by inflammation of the membranes of the cord, s mentioned by Sir B. Brodie; the patient died, and the membranes were found inflamed.

Cases of the third form, or "hysterical paraplegia," may be seen every day in the wards of this hospital. It is generally cured without much difficulty, by medicines which improve the general health, and by taking care to withdraw the attention of the patient from the part.

Paraplegia induced by great debauchery, though at first merely functional, and susceptible of cure when early attended to, if neglected soon becomes incurable, and organic change takes place; this change will probably be of the nature of ramollissement. I may here mention the post-mortem examination of a man who had formerly laboured under this form of disease.

A public-house keeper, of the name of Cutler, who had always been in the habit of drinking very freely, came under the care of Dr. Seymour about the year 1839, for paraplegia. He was cured—but some months afterwards he again came into this hospital for diseased heart and dropsy, of which he died. On opening the body, besides the disease in the chest, the brain was found to be soft and watery, there was some fluid in the ventricles, and a considerable quantity between the membranes, and in the theca vertebralis. Is it not highly probable that the paralysis was produced by the same state of the nervous system, only in a higher degree?

So far I have followed the arrangement adopted by Sir Benjamin Brodie, in a lecture on paraplegia delivered by him in the early part of last year, in this hospital. I must, however, presume to make some alteration in the organic causes.

*Organic Causes.*

1st.—Effusion of fluid into the theca vertebralis.
2nd.—Disease of the spinal cord, or its membranes, as ramollissement, tumors, &c.
3rd.—Disease in the encephalon.
4th.—Disease of the brain and spinal cord.

*1st. Effusion into the Theca.*—Very often no reason can be assigned for the presence of this fluid. Occasionally, however, the cause can be traced, as for instance, in cases of carcinoma of the vertebræ, not producing paraplegia in itself, but irritating the membrane with which it is in contact, causing this to pour out fluid, and so producing pressure on the spinal cord. Dr. Baillie supposes that it may sometimes happen that if there is any effusion of serum between the membranes of the brain, which is of very common occurrence, a portion of the serum may fall into the cavity of the theca, and press upon the lower portion of the spinal marrow.

*2nd. Disease of the Spinal Cord.*—As an example of paraplegia arising from this cause, I shall mention the case of a man of the name of Walshe, who was in this hospital in the latter part of the year 1840. Six weeks before his admission, he first perceived that the power over his hands and arms was considerably impaired; and about a fortnight afterwards, the lower extremities became affected, but not to so great a degree. On examining him in the hospital, it was found that he was unable to raise his arms more than about a foot from his body; he was also incapable of holding anything in his hand. He was still able to stand; but had some difficulty in walking across the ward. Sensation was but very slightly affected. There was no disease apparent in the vertebræ; no head symptoms. After remaining here for some time, he had an attack of erysipelas which proved fatal.

In the examination after death, the spinal marrow corresponding to the space occupied by the third and fourth cervical vertebræ, was found to be very much softened, and in a semi-fluid state. The rest of the cord was healthy. The brain was rather watery, the ventricles distended with fluid.

*3rd. Disease in the Brain.*—It is only of late years, I believe, that paraplegia has ever been referred to this cause; and many medical men still refuse to acknowledge it. In the early part of 1815 Mr. Earle published a report of a case of paraplegia in which, on examination after death, the spine was found to be perfectly healthy. In the sixth volume of the Transactions of the College of Physicians, will be found a very interesting paper on this subject by Dr. Baillie. In this, he states that, "in adults, where the spinal column has not been injured, paraplegia depends most commonly upon a disease affecting the brain itself." Dr. Baillie mentions, however, in support of this opinion, but one case, in which the pia mater was much congested, and there was considerable effusion between the membranes, and in the ventricles, the substance of the cerebellum moreover was considerably softer than natural.

This conclusion of Dr. Baillie's is probably too general, as certainly many cases of this affection arise from disease in the spinal cord alone, but at the same time it has done much good by directing the attention of practitioners in these cases not only to the spine but also to the head, the seat in very many instances of the disease.

I shall here quote, in an abbreviated form, from the valuable paper by Mr. Earle upon this subject, published in the 13th vol. of the Medico-Chirurgical Transactions, some remarks on the differences exhibited in the symptoms of paraplegia as arising from these two causes.

Paraplegia from disease in the brain, generally occurs at the middle or more advanced period of life than is usual in diseases of the bodies of the vertebræ, or of the fibro-cartilages. Its progress is more rapid, and the affection is more general, the upper as well as the lower extremities being frequently affected. The disease is more frequent in men than in women. The gait of persons labouring under cerebral affection is peculiar, and very different from that attendant on spinal disease. It nearly resembles the walk of a drunkard; they are unable to walk in a straight line, the limbs are loose, there is a great consciousness of weakness, and much difficulty in turning round. The appearance of the eyes is very similar to those of a drunkard. This analogy is readily understood, when we consider that it is the temporary disturbance of the brain from the congestion of its blood-vessels, that deprives the drunkard of the power of directing his steps, and for the time induces a state bearing the closest resemblance to paraplegia.

Sensation is more impaired than in spinal diseases, the patient appearing to feel through a false medium; the limbs are more wasted and flabby, and without that spasmodic rigidity of the muscles so common in affections of the spine. There is generally less gastric disturbance. In some instances there is the additional confirmation of an impaired state of some of the external senses, accompanied with vertigo, a sense of weight on the head, and general disturbance of the cerebral functions.

Mr. Earle quotes four cases in illustration; in the first, on examination, the vessels of the dura and pia mater were very turgid; the ventricles contained about five ounces of limpid fluid. The pons varolii and optic nerves were covered with yellowish lymph. The whole pia mater was studded with small tubercles like millet seeds; these were particularly abundant on the middle lobe and basis of the brain. The upper portion of the cord was perfectly healthy; the lower was not examined. In the second, there was much gelatinous deposit at the base of the cranium between the layers of the arachnoid; the cellular tissue of the pia mater was loaded with fluid. Three scrofulous tubercles were found in the cerebrum, and one in the cerebellum, which had suppurated; the surrounding medullary matter was soft and pulpy. The cancellous structure of the bodies of the vertebræ was filled with cheesy deposit, but no perceptible change had taken place in the spinal marrow and its membranes.

In the two remaining cases, the symptoms clearly pointed to the brain as the seat of the disease, but no opportunity was afforded of ascertaining the fact.

The cases which I shall bring forward are these:—1st. Edward Gee, æt. 38, admitted in the early part of 1841, with partial paralysis of the lower extremities, rendering his gait very unsteady, sensation but slightly impaired; had been subject to fits, preceded by giddiness and pain in the head for six months previously. Had been in the habit of drinking freely. In the course of a short time the paralysis of the lower extremities became more and more complete, till at last he was unable to quit his bed. There was great tendency to coma, and his memory became much impaired. Urine and fæces passed involuntarily. The sight became affected, the eyes appeared dull and protruding. On examination after death, the cerebrum was healthy, there was a small quantity of fluid in the ventricles. The cerebellum contained a large tumor about the size of a walnut, replacing the cerebellar structure of the anterior portion of the left lobe. The tumor was hard, homogeneous in its texture, and contained no vessels. The capsule was in part formed by the tentorium cerebelli, the rest being connected with the structure of the cerebellum by loose cellular tissue. It did not appear to press on the peduncles of the cerebellum. The pineal gland was enlarged with similar tubercular deposit. The spinal cord was perfectly healthy.

*Case 2nd.*—Anne Jackson, æt. 66, admitted for giddiness and pain at the back part of the head. She was unable to walk steadily, frequently falling down in attempting to cross the ward. Sensation not much impaired. A few days after admission she was attacked with fever, of which she died. On examination, the cerebrum was found to be vascular, but otherwise healthy; the ventricles contained a considerable quantity of fluid. On removing the tentorium cerebelli, a large tumor of about the size of an egg, was found attached to its under surface by a narrow pedicle. The tumor pressed upon and occupied the place of a large portion of the right lobe, which was considerably softened. The structure of the tumor appeared to be of a medullary character.

I have also seen two other cases of this affection, in both of which the cerebellum was the seat of the disease: in one, a lady, in whom the symptoms were peculiarly distressing, both lower and upper extremities becoming gradually paralyzed, and the sight completely lost, in which state she remained for many months; a large tumor of a malignant character was found imbedded in the right lobe of the cerebellum. In the other, where the more prominent symptoms consisted in violent and excruciating pain in the head, and convulsive paroxysms, a large serous cyst was found in the structure of the cerebellum. As, however, in these cases the spine was not examined, I shall not dwell upon them, the cases I have already mentioned, justifying me, in my opinion, in ranking disease in the brain as a cause of paraplegia.

The next cause I have put down, is, disease of the brain and spinal cord; of this I shall give one example.

*Case.*—Thomas Murphy, æt. 54, admitted with paralysis of the lower extremities of a week's duration, the left leg being attacked first. The spinal column appeared perfectly healthy. There had been no fits nor any affection of the external senses. He rapidly got worse, there was perfect palsy of the lower extremities, and entire loss of sensation from a little below the margin of the false ribs downwards. The legs were drawn up, the recti muscles of the abdomen tense and unyielding. Incontinence of urine and fæces; severe sloughing of the back. In a month he died.

On examination, some fluid was found effused at the lower part of the theca; a scrofulous tubercle of about the size of a pea, was found in the centre of the cord about opposite the 6th dorsal vertebra. The cerebrum was soft and watery, there was a considerable quantity of fluid between the membranes and in the ventricles. In the left lobe of the cerebellum, near the base, three scrofulous tumors, of a somewhat larger size than that in the spinal cord, were discovered; the scrofulous matter was in a hardened state.

I shall next briefly mention two curious and interesting cases, on the nature of which dissection could throw no light, and which, therefore, I am unwilling to class under the heads of either functional or organic, as some change might have taken place in the delicate nervous structure which the eye could not detect.

1st. Anne Cook, æt. 21, admitted with palsy of the lower extremities, sensation impaired but not altogether lost, double vision occasionally; incontinence of urine, but not of fæces. Sloughing of the back rapidly came on, but which, by the greatest care, was prevented from extending. Under the remedies employed she improved gradually for some time, becoming able to retain her urine and fæces, and to move the legs slightly when in a recumbent posture, the double vision still continued, though in a less degree. Suddenly, however, the back and hips began again to slough, the legs were drawn up convulsively close to the abdomen, vision became affected to a much greater degree, and there was violent pain in the head; she died at last worn out by the profuse discharge from the back. On examination after death, the substance of the cerebrum was firm and natural, it was perhaps rather wet when cut into. But little fluid in the ventricles. Substance of the anterior part of the middle lobe of the left hemisphere slightly softer than other parts; the cineritious substance was here of a pale colour. The cerebellum, pons varolii, crura cerebri, and optic nerves were apparently healthy. A small quantity of fluid was effused external to the dura mater in the spinal canal, and a very small quantity within the theca. Cord quite healthy.

*Case 2nd.*—Thomas Bailey, æt. 39, admitted with paralysis of the lower extremities; motion is not entirely lost but greatly impaired, sensation entirely lost at the soles of the feet, becoming less affected as you passed up the legs. Urine and fæces passed involuntarily. States that the attack commenced three weeks ago with numbness of the feet, and dulness of vision in the right eye; at present vision is perfect, and the pupils act naturally. Sloughs on the back. Soon after admission a cough, with muco-purulent expectoration mixed with blood, came on. In six weeks he died.

*Sectio cadaveris.*—The body was much emaciated. Large and extensive sloughs over both hips and sacrum. There was a small quantity of fluid in the upper part of the theca, which had apparently dripped down from between the membranes of the brain. Spinal cord apparently healthy, a slight alteration of colour and consistence, was thought to be observed about opposite the first lumbar vertebra, the change however, if any existed, was very slight. The sinuses of the brain contained a considerable quantity of dark blood. Some fluid in the ventricles. Structure of the cerebrum and cerebellum apparently healthy.

Having already, I am afraid, exceeded the time allotted by this Society for reading papers, I will now conclude, by recapitulating in a tabular form, the various sources of paraplegia as mentioned in the preceding part of the paper.

Paraplegia, then, may arise:

1st.—From disease of the vertebræ.

2nd.—From injury of the vertebræ or cord.

3rd.—From other causes.

1. *Functional.*
   - A. Over-excitement of the nervous system.
   - B. Inflammation of the membranes.
   - C. A peculiar state of nervous system, constituting one of the forms of hysteria.
   - D. Great draughts upon the nervous system, as from excessive venery.

2. *Organic.*
   - A. Effusion of fluid into the theca vertebralis.

B. Disease of the spinal cord, or its membranes.
C. Disease in the encephalon.
D. Disease of the brain and spinal cord.

3. Cause not to be discovered by dissection.

---

### MOVEABLE CALCULUS, THE SIZE OF A PISTOL-BULLET, TRAVERSING THE TRACHEA AND BRONCHIA.

A very curious case of this kind occurred in the practice of Mr. Ewart, Surgeon, of Alston, the particulars of which are as follow:—A young man, 29 years of age, had followed the occupation of a miner for eleven years, during the first nine of which, he enjoyed as good health as miners, of his standing, generally do. They are almost all affected with shortness of breath and mucous expectoration loaded with dust. About two years ago this man became affected with more decided fits of dyspnœa, attended sometimes with pain in the left side of the chest. These symptoms gradually increased in severity, especially the pain, which he assigned to a spot apparently corresponding to that part of the left bronchus situated below the arch of the aorta. This pain was aggravated by lying on the left side, or bending the body to the left. He had also fits of palpitation, and a sense of imminent suffocation, produced, as he alleged, by something rising in the lower part of the trachea, and obstructing the entrance of the air into the lungs. He continued, however, to follow his employment till the Summer of 1841, when he was seized with acute rheumatism, the thoracic symptoms still continuing unmitigated. The rheumatic fever was subdued by the usual means, after which the stethoscope detected consolidation of the upper portion of the left lung. In the beginning of October last, he was induced, one evening, to take an emetic, which operated severely. Next morning he was seized with a violent fit of coughing, which continued, until he brought up, with suffocating effort, a round ball through the rima glottidis. On examination, it was found to be a globular ball of stone, the size of a pistol-bullet, enclosed in a kind of membranous investment of inspissated mucus. It was found, when sawn into, to be composed principally of sand, and is of very compact texture.

The probability is that this stone was gradually formed by the progressive agglutination of sandy particles, while the man was, during five years, mining in a stratum of similar composition to that ejected from the lungs, and breathing an air containing silicious dust, and stagnant at the same time.

After the expulsion of the stone, the man was no longer troubled with palpitation, dyspnœa, or inability to lie on the left side. He felt a soreness, however, in that side, and a sensation which he could not well describe, in the spot originally alluded to. For a short time, his health was much improved. But latterly he is evidently declining, from tuberculous deposits in the lungs, to which he is hereditarily predisposed, and there is no doubt but that he is in an early stage of phthisis.

The stone has been forwarded to the Editors.

---

### CASE OF ABSCESS BEHIND THE MAMMA.
By MR. HENRY JAMES JOHNSON.

The Junior Editor of this Journal related this case to the Westminster Medical Society. It has been reported in the weekly Journals, from which we extract it.

A woman, residing in Rupert Street, Haymarket, applied to Mr. Johnson, with a painful affection of the left mamma, which she supposed was cancer; several surgeons had seen it, and had told her that it was a tumor of the breast, which would eventually assume a serious aspect. She was emaciated, and otherwise in a very ill state of health, and just in that peculiar condition in which scirrhous disease might be supposed likely to occur. The menstrual discharges were regular, and she had been a mother some few months previous to seeing Mr. Johnson. On examining the breast more accurately, it was found to be slightly enlarged, hard, and nodulated; manipulation gave but little pain, but she complained of occasional lancinating pains shooting through the gland, somewhat resembling those which accompany the progress of scirrhus. There was no retraction of the nipple. There was an obscure sense of fluctuation, but whatever the fluid might be, it was very deep-seated. Leeches were ordered to be applied, and some medicines were taken. Mr. Johnson determined to watch the case. In three weeks time the tumor had slightly increased in size; was more tender upon pressure; and the sensation of fluctuation beneath the skin, though somewhat more distinct, was yet very obscure. Mr. Johnson thought it might be a deep-seated abscess, and plunged a grooved needle through the substance of the breast; subsequently a lancet was thrust in, and a large quantity of pus flowed out, revealing an abscess in the loose cellular texture, between the mammary gland and the pectoral muscle. A piece of lint was introduced into the wound, and the orifice kept patent, by the uniting edges being occasionally forcibly torn asunder; a method which is a valuable one to employ in cases such as this, as in time a mucous membrane is formed, which lines the aperture, and it becomes eventually fistulous. She took tonics, sarsaparilla, cinchona, nitric acid, &c.

Mr. J. found one aperture insufficient to evacuate the abscess thoroughly, and was under the necessity of making another and a dependent counter opening. The aperture remained patent for some months, then closed. After the wound had cicatrised, it presented the appearance of a depressed scar, showing that the mamma was morbidly adherent to the deeper-seated structures.

Sir Benjamin Brodie has related a similar case, on which the late Sir A. Cooper was consulted. The mamma was extirpated. Another case was detailed by Mr. Kingdon to the London Medical Society, but the termination of that case was unfortunate.

We would observe that, when the case came under our notice, it certainly did feel very like scirrhus. The woman was thin, and the gland of the breast was expanded over the cyst of the abscess, and presented a hard, nodulated texture, which might very readily have imposed upon the surgeon.

The case is not an uninteresting one.

---

### Opening Mammary Abscesses and Buboes by Seton.
### By Mr. Henry James Johnson.

Mr. Johnson made some remarks on this subject to the Society. If there were suppuration in either mammary gland during lactation, the abscess might attain to a very large size before it would come near enough to the surface to present. In such cases one puncture was generally insufficient to evacuate the whole of the matter; or the opening must be a very large one, which, when closed, would form a large and unsightly scar: or many small punctures might be required, which would put the patient to great pain and distress; or lastly, a seton might be used. Of course if the abscess were small, or if there were two abscesses, or more, separated by a large amount of healthy glandular structure, it would be useless, or worse to employ the seton. But a large abscess, or two or more contiguous small ones are adapted for it. In using the seton in these cases, he generally proceeded in the following manner:—He made slight pressure over the abscess, to ascertain the lowest depending part of it, and at this point made a moderate incision. Through this he introduced an eyed-probe, armed with a strip of lint, or any thing of that sort. The probe was then directed to the opposite border of the abscess, through which the lint would be drawn, and subsequently left in it. By this means the abscess is completely evacuated of its contents, and it ultimately, with proper management, fills up.

We are convinced that we have occasionally in this way prevented the necessity for numerous openings in the mamma, and saved the patient from pain, and a tedious succession of abscesses.

A similar plan of treatment applies to some cases of bubo.

---

### CASE OF FATAL HÆMORRHAGE FROM THE EXTRACTION OF A TOOTH. By DAVID HAY, M.D.*

The following is another addition to the remarkable cases of this sort already upon record.

A gentleman, aged 31, on Sunday the 19th of December, suffering extreme pain from toothache, had the *dens sapientiæ* of the right side extracted. The tooth was loose and decayed, and was removed by the dentist, Dr. Roberts, with the forceps without difficulty. His mouth was instantly filled with blood; and the blood continuing, pressure was applied after touching the socket with camphorated spirit, and afterwards with oleum terebinthinæ.

In the evening, the bleeding increasing, Dr. Roberts first applied lunar caustic, and then very firm pressure. At 9, A.M., next morning, the bleeding continuing, Dr. Hay removed the compress, and examined the gum and socket carefully. The caustic had blackened the parts so much, that it was impossible for a time to discover the exact point from which the blood issued. At length he observed a stream from the inside of the gum next the cheek, and to this point he applied the actual cautery with effect;—a firm compress of lint was placed on the parts. He had also a saturated solution of acetate of lead, to make use of in case of bleeding. In the course of the day, the hemorrhage returned, and Dr. Roberts again applied the cautery, pressing it into the socket of the anterior fang of the tooth, from which he discovered the blood to be discharged immediately after its removal. In the evening the bleeding had moderated; and, as the acetate of lead appeared to have no particular power in checking it, the alum wash and cold applications were made use of in preference.

The bleeding went on more or less during the 22nd and 23d. He took frequent doses of the pulv. sulph. alum comp. with sulphuric acid, and occasional doses of the acidulated solution of sulphate of magnesia. The cold applications were continued, and pressure made by means of compressed sponge, till the evening, when Dr. Roberts inserted a portion of sponge tent into the socket of the tooth.

On the 24th, notwithstanding the sponge tent and compression, the oozing continued; and his lips having become blanched and his pulse small and frequent, it was evident that he was in a very perilous state. Mr. Nasmyth now visited him at 2 P.M. He did not think it advisable to remove the sponge tent, but rather to keep up a moderate degree of pressure over it by means of lint, and as the styptic powders nauseated, that they should be continued.

On the 25th, there was less bleeding, though the saliva which ran from his

---

* London and Edinburgh Monthly Journal. March, 1842.

mouth was reddened. The 26th and 27th he remained nearly in the same state, there being a greater disposition to bleed at night, which prevented him going to bed, and induced him to remain on a sofa in a half-erect posture. The sponge tent became raised, and as it was producing no benefit, it was removed. A portion of the skin of the lip, which had been accidentally touched when the cautery was applied, evinced the hæmorrhagic tendency, as it continued oozing blood for several days. The sloughs produced by the cautery and caustic now were gradually thrown off, and on Wednesday the 29th there was nearly a cessation of bleeding.

His strength was much exhausted—his surface pale and lips blanched—his pulse frequent, from 100 to 110, feeble, and compressible. He took the saccharine carbonate of iron in frequent doses, with sulphuric acid, and afterwards with nitric acid.

On the 30th, he complained of pain of the face and gums, which were swollen and spongy; the gums again began to bleed from various points both of the upper and under jaw. The slough was completely detached from the original site of the bleeding, and the alveolar process was felt bare with the appearance of threatened exfoliation. Camphorated spirits and oil of turpentine were occasionally applied to these spots; and the tincture of kino in water, chloride of sodium, bark and alum washes to the mouth frequently.

On the 2d and 3d of January, the spongy condition of the gums continued to increase, and near the points from which the tooth had been extracted the gum became very broad and flattened. His strength declined visibly. He tried lemon-juice, substituted Battley's solution of yellow bark for the iron, had wine, at first in moderate quantity, and afterwards in fuller measure, with animal broths, calf's foot jelly, eggs, &c.

On the morning of the 8th, he fainted when rising from the sofa, on which he still preferred remaining to being in bed; he complained also of dimness and indistinctness of vision. A solution of nitrate of silver (30 grs. to ℥j. of water) had been used to check the oozing from the gums from the 4th to the 6th; when Mr. Nasmyth substituted the acid solution of the nitrate of mercury, which appeared to be more powerful in arresting the bleeding, and occasioning the gums to contract and become paler,

Dr. Abercrombie visited him on the 9th, recommended his washing the mouth frequently with a strong solution of sulphate of zinc, and touching the gums twice or thrice a-day with pyroligneous acid, continuing the same means of holding up his strength, and augmenting his wine.

On the 10th, part of a slough was detached from the gum. At 4, P.M., there came on subsultus, &c., and at 4, A.M. of the 11th, the 22d day after the extirpation of the tooth, the patient died.

This gentleman had enjoyed very good health, and was in fuller condition than usual when his tooth was drawn. Four years previously he had a tooth extracted, when bleeding to a troublesome extent took place, but which was stopped by the application of caustic. In justice to Dr. Roberts it is proper to mention, that he was not informed of this circumstance, when he was called on to remove the *dens sapientiæ*.

---

### Abstract of a Paper on Opium-Smoking in China. By G. H. Smith, Esq. Surgeon in Penang. Communicated to Dr. Johnson by the Author.

*Mode of preparing the Opium for smoking. Causes of the Prevalence of the Habit. Mode of smoking. Description of a Smoking-shop. Effects of the Opium on the Smoker. Influence of the Habit on the Health, Vigour, and Conformation of the Chinese. Note by Dr. Johnson.*

The great extent to which this destructive vice is carried on in this island, and in the straits and islands adjacent, together with the almost utter impossibility of relinquishing the dreadful habit, when once acquired, opens an immense source of revenue to the East India Company, who monopolise the sale of all quantities of opium under a chest, as well as that of arrack, seree, toddy, bang, &c. The annual average revenue of this monopoly, or "REVENUE-FARMS," as they are called, for ten years past, has amounted to 4822*l.* sterling. But the quantity of opium smuggled is immense and incalculable. Benares opium is that chiefly used by the farmer for the preparation of "chandoo" (the composition smoked), on account of its weight and cheapness; but the consumers prefer the Patna opium, because it has a finer flavour, is stronger, and its effects more lasting.

The following is part of the mode of preparing the chandoo. Two balls are as much as one man can properly prepare at once. The soft inside part of the opium-ball is scooped out, and the rind is boiled in soft water, and strained through a piece of calico. The liquor is evaporated in a wide vessel, and all impurities carefully skimmed off, as they rise to the surface. The same process is gone through with the soft opium extracted from the ball; and all being mixed and evaporated to the consistence of dough, it is spread out into thin plates, and when cold, it is cut into a number of long narrow slips. These are again reduced to powder, re-dissolved, again evaporated, and ultimately rolled up into balls, and a good deal resemble shoemaker's wax. In this state it is fit for smoking, and is at least twice the strength of crude opium. The CHANDOO, when once smoked, does not entirely lose its powers, but is collected from the head of the pipe, and is then called "TYE-CHANDOO," or fæcal opium, which is made into pills, and swallowed by those whose poverty prevents them from smoking the CHANDOO itself.

In Penang, the opium-smokers are the Chinese, the Malays, and a very few of other nations, chiefly the native Portuguese. It is calculated that 10 per cent. of the Chinese, 2½ of the Malays, and about 1 per cent. of other natives, are addicted to the vice of opium-smoking. The poorer classes smoke in the shops erected for that purpose, but the wealthier orders smoke privately in their own houses. The practice is almost entirely confined to the male sex, a few abandoned prostitutes of the other sex partaking of the vice. A young beginner will not be able to smoke more than five or six grains of CHANDOO, while the old practitioners will consume 290 grains daily!!

The causes which lead to this dreadful habit among the Chinese are,—First, their remarkably social and luxurious disposition. In China, every person in easy circumstances has a saloon in his house, elegantly fitted up, to receive his friends, with pipes, chandoo, &c. All are invited to smoke, and many are thus induced to commence the practice from curiosity or politeness, though few of them are ever able to discontinue the vice afterwards.

Parents are in the habit of granting this indulgence to their children, apparently to prevent them from running into other vices still more detestable, and to which the Chinese are more prone than, perhaps, any people on earth. There is another cause which leads great numbers of young men into the practice of opium-smoking, a belief, founded, it is said, on experience, that the said practice heightens and prolongs venereal pleasures. It is, however, admitted by all, that opium-smokers become impotent at a much earlier period of life than others. In painful or incurable diseases, in all kinds of mental or corporeal sufferings, in mercantile misfortunes, and in other reverses of fortune, the opium-shop is resorted to as an asylum, where, for a time at least, the unfortunate may drown the recollection of his cares and troubles in an indescribably pleasurable feeling of indifference to all around. The Malays are confident that opium-smoking inspires them with preternatural courage and bodily strength; it is, therefore, resorted to whenever any desperate act is in contemplation.

The smoking-shops are the most miserable and wretched places imaginable: they are kept open from six in the morning till ten o'clock at night, each being furnished with from four to eight bedsteads, constructed of bamboo-spars, and covered with dirty mats and rattans. At the head of each there is placed a narrow wooden stool, which serves as a pillow or bolster; and in the centre of each shop there is a small lamp, which, while serving to light the pipes, diffuses a cheerless light through the gloomy abode of vice and misery. On an old table are placed a few cups and a tea-kettle, together with a jug of water, for the use of the smokers. At one side of the door the sub-farmer, or cabaret-keeper, sits, with shandoo, pipes, &c., for the accommodation of his customers. The place is filled with the smoke of the chandoo, and with a variety of other vapours, most intolerable to the olfactories of an European. The pipe, as may be seen, is composed of a shank and a head-piece, the former made of hard and heavy wood, fourteen inches long by three inches and a half in circumference. It is bored through the centre, from the mouth-piece to the head, where there is a kind of cup to collect the "TYE-CHANDOO."

The smokers generally go in pairs, and recline on the bedstead, with head resting on the wooden stool. The mode of proceeding is as follows:—First, one of the pair takes up a piece of chandoo on the point of a short iron needle, and lighting it at the lamp, applies it to the small aperture (resembling the touch-hole of a gun), in the head of the pipe. After a few whiffs he hands the pipe to his friend, who lights another piece of chandoo at the lamp; and thus they go on alternately smoking till they have had sufficient, or until they are unable to purchase any more of the intoxicating drug. The fume is always expelled through the nose, and old stagers even draw it into their lungs before it is expired.

During this time, they are at first loquacious, and the conversation highly animated; but, as the opium takes effect, the conversation droops, and they frequently burst out into loud laughter, from the most trifling causes, or without any apparent cause at all, unless it be from the train of thoughts passing through their excited imaginations. The next phase presents a vacancy of countenance, with pallor, and shrinking of the features, so that they resemble people convalescing from a fever. A dead silence precedes a deep sleep, which continues from half an hour to three or four hours. In this state the pulse becomes much slower, softer, and smaller than before the debauch. Such is the general process almost invariably observed among the Chinese; but with the Malays it is often very different. Instead of the placidity that ushers in the profound sleep, the Malays frequently become outrageously violent and quarrelsome, and lives are occasionally lost in these frightful orgies!

The CHANDOO is sometimes employed for the purpose of self-destruction; but from its strong smell and taste, it is never used as poison for others. It does not appear that sudden death is ever produced by an overdose of chandoo when used in smoking. When an inordinate quantity has been expended in this way, headach, vertigo, and nausea are the effects, and are only relieved by vomiting.

When a person has once contracted the habit of opium-smoking, he finds it extremely difficult to discontinue the vice; yet there are many instances of its being conquered by resolution of mind. In such attempts it is most dangerous to approach the opium-shops, as the smell of the chandoo produces an irresistible desire to indulge once more in the pernicious habit; neither can opium-smoking be suddenly abandoned without some substitute, as the most serious or even fatal consequences would ensue. The best substitute is a tincture of the "TYE-CHANDOO" (which is about one-fourth the strength of the "CHANDOO" itself), made with SAMSOO, a spirit made from rice, and taken in gradually diminished doses, till the habit is broken.

By a continuance in this destructive practice, the physical constitution and the moral character of the individual are deteriorated or destroyed, especially among the lower classes, who are impelled to the commission of crimes, in order to obtain the means of indulging in their dominant vice.

The hospitals and poor-houses are chiefly filled with opium-smokers. In one that I had charge of the inmates averaged sixty daily, five-sixths of whom were smokers of chandoo. The baneful effects of this habit on the human constitution are conspicuously displayed by stupor, forgetfulness, general deterioration of all the mental faculties, emaciation, debility, sallow complexion, lividity of lips and eyelids, languor and lack-lustre of eye, appetite either destroyed or depraved, sweetmeats or sugar-cane being the articles that are most relished. In the morning these creatures have a most wretched appearance, evincing no symptoms of being refreshed or invigorated by sleep, however profound. There is a remarkable dryness or burning in the throat, which urges them to repeat the opium-smoking. If the dose be not taken at the usual time, there is great prostration, vertigo, torpor, discharge of water from the eyes, and in some an involuntary discharge of semen, even when wide awake. If the privation be complete, a still more formidable train of phenomena take place. Coldness is felt over the whole body, with aching pains in all parts. Diarrhœa occurs—the most horrid feelings of wretchedness come on; and if the poison be withheld, death terminates the victim's existence.

It is generally remarked, as might, *à priori*, be expected, that the offspring of opium-smokers are weak, stunted, and decrepit. It does not appear, however, that the Chinese, in easy circumstances, and who have the comforts of life about them, are materially affected, in respect to longevity, by the private addiction to this vice, so destructive to those who live in poverty and distress. There are many persons within the sphere of my own observation, who have attained the age of sixty, seventy, and more, and who are well known as habitual opium-smokers for more than thirty years past. It is a well-known fact, that the present Emperor of China was a slave to the pernicious habit of smoking opium for many years; but that, by great moral courage and perseverance, he weaned himself from the vice, and has ever since become a most violent persecutor of those who are addicted to the indulgence. He accordingly issued edicts of severe punishment against the smoker, vendor, importer, and all concerned in the traffic of opium; and, finding these ineffectual, he made the crime capital, and punished it with death. Whatever may be said in favour of the opium traders, and against the policy or justice of the Chinese emperor, I am convinced in my own mind that the real object of his edicts was the good of his subjects, and that he hoped, however vainly, to eradicate a vice destructive alike of the health and morality of those who become its victims. But his Majesty's government acted on very different principles; namely, the most selfish, venal, and mercenary. It is a notorious fact, that many, perhaps most of the officers, employed in preventing the importation and smuggling of opium, are themselves opium-eaters, or opium-smokers, and consequently that they wink at the illicit trade, or take bribes of opium or dollars for the introduction of the drug. It is well known now, that in several of the southern provinces of China, opium is cultivated to a great extent, without any check from the local authorities, and, doubtless, without any knowledge of the emperor himself. The propensity to opium-smoking is becoming so universal and so irresistible in China, that no sumptuary laws, however sanguinary, will be able to stem the torrent. In Penang excessive duties have only increased the thirst for opium; and what is worse, they have quadrupled the number of murders and other crimes committed in order to obtain the means of procuring the drug!!

Pulo Penang, Straits of Malacca.

---

NOTE BY DR. JOHNSON.

The foregoing paper has been laid before the society, partly because the subject is curious, and little known in this country, but chiefly for the purpose of offering one or two practical suggestions to the members.

First, I think it will be admitted that the Chinese mode of taking opium by smoking or inhalation, induces the peculiar sedative effects of that drug more powerfully and more speedily than when taken into the stomach.

Second, There can, I believe, be little doubt, that these effects are produced chiefly, if not entirely through the medium of the nervous system, and not by digestion, absorption, and the circulation.

Third, it does not appear that the casual or temporary smoking of opium is more dangerous or injurious to the constitution than that of swallowing the drug, whether in substance or solution. On the contrary, I believe it is less so, and not so likely to impair the functions of the stomach, liver, and bowels, as when directly applied to the digestive apparatus.

Fourth, The *habitual abuse* of a drug, by which, in fact, it is converted into a poison, is no argument or reason against its occasional exhibition as a remedial agent.

Fifth. If the above observations be admitted as rational, I see no reason why we should not employ the Chinese mode of inhaling the fumes of opium, in certain dangerous and painful maladies where the common mode is found to be inefficient, and attended with great derangement of the digestive organs. It is clear that we can very seldom induce that profound sleep and insensibility to all mental misery and corporeal pain, by opium taken into the stomach, which we find to be produced by the inhalation of its fumes acting directly on the brain, through the medium of the nerves. Might not the Chinese mode, then, be adopted in tetanus, hydrophobia, tic-douloureux (especially of the facial nerves), violent spasms, and painful diseases that defy the power of opium taken in the common way?

The various preparations of opium might be easily smoked by means of a common pipe, and the powerful effects induced in a very short space of time, without the possibility of their being rejected by the stomach, or prevented from acting energetically on the sensorium, and throughout the whole nervous system.

---

A Suggestion for the Cure of Cholera. By Lieut. H. Congreve, Madras Artillery. Richardson, London.

The disease which, ten years ago, frightened this Isle from its propriety, is now as seldom thought of as the Spanish Armada, or the Norman Conquest. Yet, like Sir R. Peel's bad harvests, it may come again—in cycles—and then all the old remedies, that proved no remedies at all, will be furbished up, and perhaps the " Suggestion " of Lieutenant Congreve may have its day of trial. Our author's theory—for it is no more—may be briefly stated. He considers that cholera " is propagated by the transmission of the poisoned air from the lungs of the person who has inhaled the noxious gas, to another individual, who, in like manner, disseminates the distemper by infecting the atmosphere which those he comes in contact with may breathe." To this he adds, that " the poison exhaled from a diseased person may remain suspended in the air for a considerable space of time, and still be productive of fatal results to those who subsequently inhale it."

We need not say how totally inadequate this hypothesis is to explain the *propagation* of cholera. Of the *nature* of the poisonous gas, Mr. Congreve does not pretend to judge; but he " hazards the opinion that it usurps the place of oxygen in its combination with nitrogen to form the air." The remedy naturally flows from the supposed cause—it is the inhalation of oxide of nitrogen—which has the " singular effect of producing a high degree of excitement, after a few inspirations." An oil-silk bag, capable of containing several quarts, is necessary. If this cannot be procured, a large bladder may be employed. But the gas is not so easily obtained on the spur of the moment, and in the midst of sickness and terror.

We think we have heard of this proposal before, but cannot say where.* We

---

* Since writing the above, we hear that Mr. Martin, of Calcutta, exhibited oxygen gas in 1827, to a patient labouring under cholera—in the last stage of collapse—with remarkable good effects for half an hour; but from want of shall therefore give Mr. Congreve the credit of priority. We wish sincerely that oxygen gas may be found a cure for cholera; but as yet there is no proof. It is, as the title-page of the pamphlet denominates it—a " SUGGESTION."

---

### OBSERVATIONS ON THE MINERAL SPRINGS OF HARROGATE. By Dr. BENNETT.

This small brochure has accidentally fallen into our hands. It is the first of a series on the same subject. In this, Dr. B. has merely examined the temperature and the solid contents of four of the Harrogate Springs, during the different months of the year—from 14th April, 1841, till the 2d of March, 1842. From these tables it appears, that both the temperature and the aggregate of solid contents vary considerably at different times of the year. Thus the Old Sulphur Well varied from 53° down to 40°—the highest temperature being in September, when the atmosphere was 62°—and the lowest 40° in February, the air being 43°.

It is therefore evident that the temperature of the water corresponded pretty much with that of the air, and consequently that the sources were at no great distance from the surface.

In respect to the aggregate solid contents of the Old Well, it varied from 54 grains (in eight ounces) in the month of April, down to 45 in the month of October. This variation was probably owing to the prevalence of rains or droughts.

Dr. Bennett and others have observed that, where the sulphur waters were heated, they sometimes acquired a greenish or rather yellowish hue. This was attributed to the stone jars in which the waters were heated. This is proved to be erroneous—for Dr. Bennett found that water heated in glass vessels assumed the same hue at times. Dr. B. attributes the yellow hue to *lime* in the water, which is sometimes more—sometimes less copious. He thinks the phenomena should be obviated by a suitable apparatus, in which the water may be heated to 85 or 95 degrees, where the cold water cannot be borne.

We shall look, with interest, to the further pursuit of Dr. B.'s investigations—more especially those which relate to the medicinal agency of the Harrogate waters.

One of the greatest recommendations—perhaps virtues, of the cold continental mineral waters, is their large impregnation of carbonic acid gas, which makes many of them as brisk as Champaigne, and, consequently, very agreeable to the palate. In the case of chalybeates—perhaps in all the saline springs, also, the said carbonic acid dissolves the iron more completely, and renders it more efficacious than when diffused in flat and insipid waters. Thus the springs of Spa, Schwalbach, Bruchenau, Franzensbad, and of many other places on the continent, present a contrast, in respect to carbonic acid gas, with Tunbridge Wells, or other chalybeate waters in this country. The idea, therefore, of impregnating the native chalybeate waters of England with carbonic acid, is a good one—and has been commenced (under the direction of Dr. Bennett, we believe), at Harrogate. The Tewitt Spring there, a very good chalybeate, has been selected for the process of aeration, and Mr. Clayton, chemist, of Upper Harrogate, has sent us some bottles of the aerated water, which is as brisk as soda water, and reminds us of the Weinbrunnen, the Bruckenau, and the Franzenquelle. We think the process might be advantageously applied to some of the other saline waters at Harrogate, by which their taste, and perhaps their medicinal agency might be considerably improved.

---

proper apparatus, was unable to continue the experiment. See Influence of Tropical Climates, &c. 6th edition, 353. By Dr. Johnson, and Mr. Martin.

A Pentaglot Dictionary of the Terms employed in Anatomy, Physiology, Pathology, Practical Medicine, &c. &c. &c. In Two Parts. Part I. With the leading Term in French, followed by the Synonymes in the Greek, Latin, German, and English, &c. &c. Part II. A German-English-French Dictionary, &c. &c. By Shirley Palmer, M.D. 8vo. pp. 656, in double columns, small type. Longman and Co. 1841.

In this stupendous work we scarcely know which to admire most—the extensive erudition, or the unwearied, we might say super-human, labour of its author! Dr. Palmer may well exclaim with the Roman bard—

"Exegi monumentum Ære Perennius."

Johnson's Great Dictionary in four volumes cost not one-tenth the pains and research that Dr. P.'s Pentaglot must have done. Dr. Palmer will not be rewarded during his life-time for the labour he has undergone, and the wear and tear of mind and body which he must have experienced in the construction of a book that might well be considered a hard task for a long life of literary drudgery in the study, free from every other avocation or pursuit. What then must have been the destructive toil by the midnight lamp, stolen from rest and sleep, during the compilation of this immense cyclopædia of dry technical terms, definitions, and derivations?

Any analysis, or even the most superficial review of such a performance would be preposterous. The only thing we can do is to take a specimen at random—a brick out of a majestic piece of architecture.

"Anévrysme, Aneurysme, s. m.,—*ἀνεύρυσμα* (*ἀνευρύνω*, I dilate),—aneurisma, aneurysma, n. L.,—aneurisma, anevrysma, n., pulsadergeschwullst, f., die erweiterung einer arterie, G.,—aneurism, swelling, dilatation of an artery. Aneurism may be defined, a tumour, formed by arterial blood, from dilatation, rupture, or division, of the coats of an artery. The term has been also applied, by some writers, to dilatation of the cavities of the heart, and even to enlargement of the organ from thickening of its parietes.

Aneurism shews itself under three different forms: 1. that of *true* aneurism, —*vrai*, F.,—aneurysma *verum*, L.,—das *wahre* anevrysma, G.,—formed by dilatation, circumscribed or diffused, without breach, of all the coats of an artery. In the *former* case, it constitutes the variety called *circumscribed*,—circonscrit,—circumscriptum,—umschreibene;—in the *latter*, the *diffused*,—diffus,—diffusum, ausgebreitete,—of *true* aneurism: 2. *false* or *spurious*,—*faux*,—*spurium*,—das *falsche* anevrysma,—formed by a breach of two or all of the arterial tunics, and presenting two varieties: the *circumscribed*, in which the blood, escaping through a rupture of the internal and middle coats, converts the external coat of the vessel into an aneurismal sac;—and the *diffused*,—where the external coat, also, has subsequently given way, and the blood been poured out into the surrounding cellular structure: 3. *mixed* aneurism,—*mixte*,—*mistum*,—das *gemischte*—which likewise comprehends two varieties; one, the *internal*, consisting of an hernia-like protrusion of the internal, through a wound or rupture of the middle and external coats of an artery;—and the other, *external*, produced by rupture of the dilated coats of true aneurism, and consequent diffusion of its contents through the circumjacent membrane.

Besides these principal forms, there is *Aneurism by Anastomsis*,—anévrysme par anastomose, F.,—das anastomotische anevrysma, G.,—apparently caused by aneurismal dilatation of the extreme vessels of a part, and extravasation of blood into the distended cells of the cellular structure."

We need hardly say that a work like this, which costs but a trifle, though the result of years of labour, should be in the hands of every student and practitioner who wishes to keep pace with the current of medical literature, and glean knowledge from foreign as well as domestic sources.

